# Uncertainty in Blood Pressure Measurement Estimated Using Ensemble-Based Recursive Methodology

**DOI:** 10.3390/s20072108

**Published:** 2020-04-08

**Authors:** Soojeong Lee, Hilmi R Dajani, Sreeraman Rajan, Gangseong Lee, Voicu Z Groza

**Affiliations:** 1 Department of Computer Engineering, Sejong University, 209 Neungdong-ro, Gwangjin-gu, Seoul 05006, Korea; 2 School of Electrical Engineering and Computer Science, University of Ottawa, Ottawa, ON K1N6N5, Canada; hdajani@uottawa.ca (H.R.D.); Groza@eecs.uOttawa.ca (V.Z.G.); 3 Department of Systems and Computer Engineering, Carleton University, Ottawa, ON K1S5B6, Canada; sreeramanr@sce.carleton.ca; 4 Ingenium College, Kwangwoon University, 20 Kwangwoon-ro, Nowon-gu, Seoul 01897, Korea; gslee0115@gmail.com

**Keywords:** uncertainty, confidence interval, oscillometry blood pressure measurement, deep neural network, ensemble method

## Abstract

Automated oscillometric blood pressure monitors are commonly used to measure blood pressure for many patients at home, office, and medical centers, and they have been actively studied recently. These devices usually provide a single blood pressure point and they are not able to indicate the uncertainty of the measured quantity. We propose a new technique using an ensemble-based recursive methodology to measure uncertainty for oscillometric blood pressure measurements. There are three stages we consider: the first stage is pre-learning to initialize good parameters using the bagging technique. In the second stage, we fine-tune the parameters using the ensemble-based recursive methodology that is used to accurately estimate blood pressure and then measure the uncertainty for the systolic blood pressure and diastolic blood pressure in the third stage.

## 1. Introduction

Blood pressure (BP) always fluctuates due to factors such as stress, exercise, disease, and inherent physiological oscillations [1]. However, the physiological variance of BP, which can rise up to 20 mmHg, has been neglected so far [2]. Even though physiological uncertainty is larger than the margin of error of standard BP measurement protocols. The issue of accuracy, precision and uncertainty in the measurement of physiological parameters has been of constant concern for practitioners [3]. Even though the standard for the expression of uncertainty in measurement [4] states that it is applicable to a broad span of fields, in practice it has been applied only to measurements determined on the basis of a series of observations obtained under repeatable conditions, a situation which is rarely reproducible in physiological measurements. Recently cuff-less BP devices using the photoplethysmograph (PPG) sensors have recently been used to measure BP [5,6,7]. Kachuee et al. [5] proposed a method to estimate BP based on the PPG for continuous health-care monitoring. A continuous BP estimation method using machine learning was introduced by Chen et al. [6]. Tjahjadi et al. [7] a novel classification method for BP based on PPG signal using long short-term memory (LSTM). However, automated oscillometric blood pressure measurement methods [8,9] are more commonly and popularly used to measure BP for many patients at the home, office, and medical centers. These devices usually offer a single BP value. However, these devices are unlikely to provide values that are superior to those that may be obtained by repeated BP measurements [10]. That is because individual BP measurements are inherently subject to sources of uncertainty sources that cause deviations of the measured value (i.e., estimate) from the true BP value (i.e., reference BP) [11]. The sources of uncertainty can be separated into random errors and systematic errors [11], which will be dealt with in more detail in the following section. If BP measurements are simultaneously influenced by many sources of uncertainty, the distribution function of these measurements converges towards Gaussian distribution as the number of uncertainties increases, regardless of the distribution function of parameters representing the source of uncertainty [11]. Few researchers have tried to study uncertainty in physiological measurements [12,13] and there have been no attempts to include the characterization of the quality of the acquired signal and its compatibility with the employed estimation algorithms in a global figure of merit of confidence in the measurement accuracy. Thus, the confidence interval (CI) should be provided in such a way as to assess and express uncertainty in BP measurements where CI provides an estimated range of BP values, which possibly includes significant unknown sources [11]. Based on some aggregated statistics, wide CIs can provide alerts to patients, medical staff, and families. Thus, measuring the CI of a blood pressure measurement is very important, but unfortunately, there has been very little study done to determine the CI for an oscillometric BP measurement. Indeed, in order to estimate the CI for each patient, we need many BP measurements. However, it is very difficult to measure BP multiple times for each patient using an oscillometry BP device since repeatable circumstances for reproducible BP measurements cannot be guaranteed [14]. For this reason, it is necessary to calculate CI using a small number of measurements, and as a result, the bootstrap technique was proposed to obtain CI estimates from BP measurements using a small sample size [14].

Soueidan et al. also proposed a new method to augment the noninvasive measurement by providing the mean systolic blood pressure (SBP) and diastolic blood pressure (DBP) with CIs [15]. However, these methods did not satisfy the allowable bias specified by the standard protocol [16]. To address this issue, Lee et al. recently introduced the deep neural network (DNN) estimator [17] to estimate BP measurements. However, this method has many random and initialized parameters in the training procedure, such as weights and bias, which can cause unstable estimates such as large standard deviations of errors. To address the above challenge, Lee et al. also provided a method to obtain accurate BP estimates using the DNN ensemble estimator [18]. Here, we introduce a novel methodology using an ensemble-based recursive methodology (EBRM) to measure uncertainty for oscillometric BP measurements. There are three stages to the methodology: the first is pre-learning to initialize good parameters using the bagging technique [19], where the sample number of artificial features is augmented using the bagging technique to obtain effective ensemble parameters by training each estimator. After that, in the second stage, we fine-tune the parameters using the EBRM that is used to accurately estimate BPs and then measure the uncertainty for the SBP and DBP in the third stage. As far as we know, the proposed method is one of the first studies using the EBRM based on the DNN model to measure the uncertainty for the SBP and DBP. This paper is an expanded version of the paper [14,18] with the following contributions:

- The proposed methodology can measure uncertainty such as CIs, the standard deviation of error, bias, standard uncertainty, and expanded uncertainty for the SBP and DBP.
- We provide the standard uncertainty *u*, the combined uncertainty $u_{c}$, and the expanded uncertainty *U* and all are computed based on the approaches detailed in GUM [4] using the bias and standard error for artificial features for the SBP and DBP.
- The previous estimated SBP and DBP are also initialized as another input matrix for the EBRM with the DNN model. This is a novel method as the EBRM is different from the conventional AdaBoost technique.
- We execute Lilliefors test to validate that the distribution of the artificial BP features approaches the Gaussian distribution and to identify similarities between the actual data and the artificial data.

The introduced methodology is expressed as follows. First, we acquire the features from the oscillometric waveform (OMW) signals and envelopes after pre-signal processing on the BP signals. We then create the artificial features from the original features and evaluate the normality of the distribution of all the features. We then constitute the proposed EBRM through the pre-training and a fine-tuning [17]. Based on the EBRM, we estimate the BP values (SBP and DBP) for the individual subjects. Subsequently, to measure the CIs, standard deviation of error, bias, standard uncertainty, and expanded uncertainty for the SBP and DBP. We then confirm the normality of the artificial BPs for individual subjects.

## 2. Methods

### 2.1. BP Measurement and Protocol

The study was approved by the institutional ethics committee, and each test subject provided informed consent. The BP data were measured from 85 people who do not have cardiovascular disease, aged 12 to 80 years with 48 men and 37 women. Particularly, the mean age of 85 subjects was 40.4, the standard deviation was 15.2 and there were six people under 19 in all subjects. A wrist-mounted blood pressure device was used to obtain five sets of oscillometric BP measurements using a piezoelectric sensor embedded in a pressure cuff from each subject following the American National Standards Institute (ANSI)/Association for the Advancement of Medical Instrumentation (AAMI) protocol [16,20]. The average value measured by the two trained observers was used as the reference value for SBP and DBP [14]. This process was repeated four more times to generate five sets of BP data for each subject, with a one-minute break between each BP measurement. Each subject comfortably sat on a chair during the measurement, with a BP cuff wrapped around the subject’s left wrist and comfortably raised her or his arm, which was raised to heart level. For the reference measurement, an auscultatory BP cuff was worn at the top of the left arm to match the height of the heart. Notably, two nurses applied a blood pressure cuff to the patient’s upper arm. Then, the pressure was increased on the cuff to block the brachial artery. Hence, the blood flow generated a Korotkoff signal (KS) that was heard with the aid of a stethoscope. The first KS, which was determined in units of mmHg by a manometer connected to the upper cuff, was used to predict the SBP, while the fifth KS was used to predict the DBP. However, it was impossible to measure the blood pressure of the arm and wrist at the same time because of the occlusion problem of brachial arteries by arm sphygmomanometers. Hence, almost 1.5 min after each pulse wave signal acquired by the wrist BP monitor, two trained nurses concurrently recorded (${\mathrm{SBP}}_{1}$) and (${\mathrm{SBP}}_{2}$) and (${\mathrm{DBP}}_{1}$) and (${\mathrm{DBP}}_{2}$) using a upper arm sphygmomanometer. The first and second nurses concurrently obtained the BP readings. Hence, the results of five sets were given by (${\mathrm{SBP}}_{1i},{\mathrm{SBP}}_{2i},\left|i=1,...,5\right)$ and (${\mathrm{DBP}}_{1i},{\mathrm{DBP}}_{2i},\left|i=1,...,5\right)$ for each subject, respectively, where subscript 1 and 2 denoted the first and second nurses. Therefore, each of five classic arm sphygmomanometer measurements obtained by the first and second nurses as $\left[\left({\mathrm{SBP}}_{1i},{\mathrm{SBP}}_{2i}\right),\left({\mathrm{DBP}}_{1i},{\mathrm{DBP}}_{2i}\right),|i=1,...,5\right]$ corresponded with an interval of 1.5 min to each of the five pulse wave signal obtained by the automated oscillometric BP device (OBPD), as $\left({\mathrm{OBPD}}_{i}|i=1,...,5\right)$ for each subject as shown in Table 1. This interval between traditional arm and wrist measurements was not only as short as possible to minimize natural BP variability over time, but could be long enough for the system to stabilize after the occlusion of arteries during measurements. The approximately 1.5 min interval between arm and wrist measurements was selected by compromise to minimize method errors [12].

### 2.2. Features Obtained from Oscillometric Signals and Artificial Data Obtained Using Bootstrap Technique

In order to estimate the reference BP value, we removed outliers using a signal processing technique and the effective features of the oscillometric waveform (OMW) signals were extracted [21]. Because the five BP data for individual volunteers represent small amounts of input data for the training process, we used the bootstrap method [22] to increase the amount of data for each volunteer, where this data is referred to artificial data or features in this study. The artificial input data were generated using the bootstrap technique [14,22] to improve estimation accuracy with traditional approaches when the datasets are limited. More details regarding these features can be found in [21].

### 2.3. Lilliefors Test for Artificial Data

The normality assumption is the key to a majority of standard steps [23]. We thus verify the normality of the artificial feature. The Lilliefors test is executed to evaluate the normality of each artificial feature as well as to correct the Kolomogorov–Smirnov test of goodness of fit for small values at the tails of probability distributions [24]. Here, we assume that $D^{*}$ is a probability distribution for an artificial feature $\left(\mu_{1}^{*},...,\mu_{B}^{*}\right)$, where *B* denotes the size of replication. We measure the homogeneity between the Gaussian distribution hypothesis and the distribution of artificial features [20]. If the Lilliefors test returns a decision value for the null hypothesis then the artificial feature comes from a normal distribution family, against the alternative in which it does not come from such a distribution. The result *h* denotes one if the test rejects the null hypothesis at the 5% significance level, and zero otherwise. Thus, we accept the null hypothesis as shown in Table 2. It is also noted that all *p* values in the Lilliefors test are greater than α (=0.05) and that null hypothesis is rejected if the Lilliefors test value *k* is larger than the critical values *c*. Therefore, we can accept the null hypothesis that the distribution of artificial features converges to the normal distribution [25]. We examine the consistency and convergence for the artificial data [22]. Therefore, we verify that our artificial data are suitable for actual data convergence for sample means based on the theorem [26] in that if $E(x^{2})<\infty$, then $\|D^{*}\left\{\sqrt{n}\left({\hat{\mu }}^{*}-\hat{\mu }\right)\leq x\right\}-D\left\{\sqrt{n}\left(\hat{\mu }-\mu \right)\leq x\right\}{\|}_{\infty }⟶0$, where X is the actual feature and ${\left\|\cdot \right\|}_{\infty }$ is $\sup_{x\in R}\left|\cdot \right|$, respectively. It is found that the distribution of $\sqrt{n}\left({\hat{\mu }}^{*}-\hat{\mu }\right)$ approximates to $\sqrt{n}\left(\hat{\mu }-\mu \right)$ [26], where $\beta \left(\mu \right)=E(\hat{\mu }\left(X\right)-\mu )$ denotes a bias and μ is the original feature. When the bias approaches zero, estimates are considered to be unbiased and we can easily compute uncertainties such as the bias and standard error for the artificial features as shown below:

(1) $$\begin{array}{cc} \beta ({\hat{\mu }}^{*}\left(\cdot \right))= & \frac{1}{N}\sum_{b=1}^{N}{\hat{\mu }}_{b}^{*}-E\left(\mu |X\right)\\ ≅ & E\left({\hat{\mu }}^{*}\left(X^{*}\right)-\hat{\mu }\left(X\right)\right) \end{array}$$

(2) $${\hat{\sigma }}^{*}=\sqrt{\left(\frac{1}{N-1}\sum_{b=1}^{N}{\left({\hat{\mu }}^{*}-{\hat{\mu }}^{*}\left(\cdot \right)\right)}^{2}\right)},$$

where $\mu^{*}\left(\cdot \right)$ denotes $N^{-1}\sum_{b=1}^{N}{\hat{\mu }}_{b}^{*}$.

## 3. Ensemble-Based Recursive Methodology (EBRM) for Measured BP

### 3.1. EBRM with DNN Regression

Here, we present the EBRM based on the bagging [19] and AdaBoost [27] techniques used with the DNN model [18]. There are three parts: the first part is pre-learning to initialize good parameters using the bagging technique, and the second part is to fine-tune the parameters using the recursive AdaBoost technique. Our input features are given as (VT), where V and T denote the input matrix and the output matrix, respectively. Then, the mean μ and standard error σ are computed for each feature vector. The bootstrap method is then used as a generator to build the distribution for artificial feature, as described in Section 2. Indeed, the Gaussian–Bernoulli restricted Boltzmann machine (GBRBM) is used to connect between a Gaussian input layer and a binary hidden layer because the distribution of artificial feature approaches a normal distribution [17]. The GBRBM [28] is utilized to mitigate the local minima problem and the issue of overfitting through the choice of initial parameters. However, random initialization parameters such as training data sets, weights, and biases at the pre-learning step are sources of uncertainty. Hence, ensemble parameters are used to solve random initialization parameter problems, and they are used in fine-tuned steps. The parameter matrix $W\in R^{m\times n}$ is built based on the given input features matrix V. We implement the bagging technique in order to minimize the standard deviation of the error of the difference between the input data and reconstructed data (i.e., estimated input data) using the Kullback-Leibler divergence [28]. Therefore, the ensemble parameters can be given as ${\tilde{W}}_{\varphi }=\sum_{k=1}^{K}W_{k}\left(\cdot \right)$, where *K* denotes the number of ensemble.

On the other hand, we acquire each artificial feature after the adaptive adjustment of the distribution of training data sets using the EBRM in the second step, as presented in Algorithm 1. A weight vector $w_{m}^{\left(1\right)}$ is initialized to be used in the second step. We then create a different training set for each estimator according to the weighted sample, *M* from the sequence of *L* with M≤L, where *L* denotes the number of artificial samples (L=B×S). Note that, in L=B×S, *B* and *S* denote number of replication and the number of subjects, respectively. Note that *I* in Algorithm 1 denotes the number of features, as shown in lines 4–8. In detail, the artificial samples are obtained from different distributions, which are updated repeatedly through relative errors and estimated BPs to be used in the next estimator. The weight of each instance is updated based on an error. In other words, it is more likely that an instance with a large error in the previous distribution exists in the next distribution as shown $\left(V^{*}|w_{m}^{\left(k\right)}\right)$ in line 7. Estimate BPs (SBP and DBP) are also initialized as another input matrix ${\hat{T}}_{m}^{\left(1\right)}$ for training EBRM with the DNN model. The estimate BPs are thus concatenated to an artificial block matrix as $\left[{\tilde{V}}^{*}\;{\hat{T}}_{m}^{\left(k\right)}\right]$ and are updated recursively as shown in algorithm line 8, where ${\hat{T}}_{m}^{\left(k\right)}$ denotes the previous BPs’ estimator. This is a novel method as the EBRM is different from the conventional AdaBoost approach [27].


**Algorithm 1**

 **procedure** EBRM(V, T)2: **for** k←1,K **do** $w_{m}^{\left(1\right)}$ and ${\hat{T}}_{m}^{\left(1\right)}$   **for** i←1,I **do**
4:    **for** b←1,B **do** $V_{i,b}^{*}=\left(v_{1}^{*},v_{2}^{*},...,v_{N}^{*}\right)$ and $T_{j,b}^{*}=\left(t_{1}^{*},t_{2}^{*},...,t_{N}^{*}\right)$,      ${\bar{V}}_{i,b}^{*}=\frac{1}{N}\sum_{n=1}^{N}v_{n}^{*}$ and ${\bar{T}}_{j,b}^{*}=\frac{1}{N}\sum_{n=1}^{N}t_{n}^{*}$,6:     $V_{i}^{*}=\left({\bar{V}}_{i,1}^{*},{\bar{V}}_{i,2}^{*},...,{\bar{V}}_{i,B}^{*}\right)$ and $T_{j}^{*}=\left({\bar{t}}_{j,1}^{*},{\bar{t}}_{j,2}^{*},...,{\bar{t}}_{j,B}^{*}\right)$      ${\tilde{V}}^{*}=\left(V^{*}|w_{m}^{\left(k\right)}\right)$
8:     $U^{*}=\left[{\tilde{V}}^{*}\;{\hat{T}}_{m}^{\left(k\right)}\right]$
     **end for**
10:  **end for**
   call learning: back-propagation $\left\{{\hat{f}}^{*k}\left(U_{m}^{*},T_{m}^{*}\right)\right\}$,12:  output: ${\hat{T}}_{m}^{*}$, ∀m=1 to *M*   $\varepsilon_{\max}=\max_{m=1,...,M}{\left[{\hat{T}}_{m}^{*}-T_{m}^{*}\right]}^{2}$
14:  $\varepsilon_{m}=\frac{{\left[{\hat{T}}_{m}-T_{m}^{*}\right]}^{2}}{\varepsilon_{\max}}$
   $\bar{\varepsilon }=\sum_{m=1}^{M}\varepsilon_{m}w_{m}^{\left(k\right)}$
16:  $\beta_{k}=\frac{\bar{\varepsilon }}{1-\bar{\varepsilon }}$
   $w_{m}^{\left(k+1\right)}=w_{m}^{\left(k\right)}\beta_{k}^{\left(1-\varepsilon_{m}\right)}$
18:  $w_{m}^{\left(k+1\right)}=\frac{w_{m}^{\left(k+1\right)}}{\sum_{m}w_{m}^{\left(k+1\right)}}$
  **end for**
20: **end procedure**


Back-propagation with scaled conjugate gradients is then executed to optimize the parameters as shown in line 11. In turn, we repeatedly calculate the error between the hypothesis $\left({\hat{T}}^{*}\right)$ and reference $\left(T^{*}\right)$ until the minimum value is reached as expressed in line 12. Here, the estimated $\left({\hat{T}}^{*}\right)$ BPs are used as an input feature to train the recursive ensemble estimator. If the error is smaller, the artificial block matrix for the next estimator in EBRM is closer to the reference BPs $\left(T^{*}\right)$. The mean error is computed as shown in lines 13–15, and the weight parameter $\beta_{k}$ is represented as in line 16. Finally, we update the weight vector for instances and normalize them as shown in steps 17–18. If the error values for each instance are very small, the weight parameters also have a small value, respectively, and if the error values for each instance are large in the current iteration, $\beta_{k}$ also have large values. The output of the EBRM is given as follows: ${\hat{T}}^{*}\left(x\right)=\inf\left\{t\in T:\sum_{k:{\hat{t}}_{k}\leq t}^{}\log\left(1/\beta_{k}\right)\geq 1/2\sum_{k}^{}\log\left(1/\beta_{k}\right)\right\}.$ Here, each *k* estimator predicts ${\hat{t}}_{k}$, ∀k=1,...,K. If $\beta_{k}$ is all equal, it will be equivalent to the median. We add up the log until we reach the smallest *k* so that the inequality can be satisfied [27]. The EBRM with DNN model is utilized as a stable estimator given as ${\hat{f}}_{\varphi }^{*}\left(\cdot \right)=D_{NN}\left(\left(U^{*},T^{*}\right)\right)\left(\cdot \right):R^{I}\to R^{J}$. In this study, $D_{NN}\left(\cdot \right)$ is the DNN model which is used as a weak learner.

### 3.2. DNN Model [28]

Our deep learning model consisting of two hidden layers is a probability-generating structure [28]. The deep learning model is given by $P\left(v^{*},h^{1},h^{2},...,h^{l}\right)=P\left(v^{*}|h^{1}\right)P\left(h^{1}|h^{2}\right)\cdot \cdot \cdot P\left(h^{l-2}|h^{l-1}\right)P\left(h^{l-1},h^{l}\right)$, where in $P(h^{i}|h^{i+1})$, $h^{i}$ denotes the hidden units at layer *i*, and $v^{*}$ is the re-sampled input data (artificial data). We can write a probability as $P\left(v^{*},h\right)=\frac{1}{U}\exp^{-h^{\prime }Wv^{*}-c^{T}v^{*}-b^{T}h}$, where *U* denotes the normalized constant value, c and b are the bias for the input data and the bias for the hidden units, respectively, and W denotes the weight parameter. We thus rewrite the conditional layer as $P\left(h^{i}|h^{i+1}\right)=\mathrm{sgm}\left(-c-\sum_{k=1}^{n^{i+1}}Wh_{k}^{i+1}\right)$, where sgm(x) denotes a sigmoid function. Looking at the data learning process, we use pre-learning to make the initial values for weights and biases efficiently, and use it as an effective point to fine-tune [29]. Thus, the cost function [17,30] can be obtained as

(3) $$L\left({\tilde{W}}_{\varphi },c\right)=\frac{1}{C}\sum_{c=1}^{C}\sum_{d=1}^{D}{\left({\hat{T}}_{c}^{*d}\left(\tilde{W},c\right)-T_{c}^{*d}\right)}^{2}$$

where $T_{c}^{*d}$ represents the $d^{th}$ target BP data (referred to as TSBP* and TDBP*) at the sample index *c*; *D* and *C* denote the size of data and the size of batch, respectively. Next, we iteratively renew the parameters as

(4) $$\left({\tilde{W}}_{\varphi (c+1)}^{i},c_{c+1}^{i}\right)=-\zeta \frac{\partial L}{\partial \left({\tilde{W}}_{\varphi (c)}^{i},c_{c}^{i}\right)}+\eta \left({\tilde{W}}_{\varphi (c)}^{i},b_{c}^{i}\right),1\leq i\leq K+1,$$

where ζ, η, *K* and K+1 denote a learning rate, a momentum parameter, the number of hidden layers and the output layer, respectively.

## 4. Uncertainty Estimation

### 4.1. Measurement Uncertainty

The quantity that we intend to measure is called measurand [11]. The aim of a measurement is to acquire the true value of the measurand. We do not know exactly how close the measured BP value is to the true BP value. Therefore, our estimates always have some uncertainty associated with it. The difference between the measured BP value and the true BP value being measured is called an error. The errors of BP can be thought of as consisting of two parts: BP random errors and BP systematic errors. We do not know BP error since true BP value is not known. Therefore, it is impossible to indeed use the quality characteristics of the BP measurement results. The quality and accuracy of the BP measurement results are characterized by uncertainty of BP measurement, defining the interval around the measured BP value, where true BP value exists with some probability. Uncertainty of BP measurement *U* itself is half-width of that interval and is always positive [11]. This uncertainty is considered to be as the estimate that is the highest probable absolute difference between the measured BP value and the true BP value.

Random errors result in differences among repeated BP measurement results. However, the more repeated BP measurements are made, the less likely the mean value is to be affected by the random errors. Thus, the influence of random errors can be reduced by increasing the number of repetitions in BP measurements. On the other hand, systematic errors cause deviations in the same direction by the same size of all BP measurements in the series. Increasing the number of iterations does not reduce the influence regarding system errors such as bias [11]. One of the most common ways to improve the measurement reliability is to make the same amount of repeated BP measurements. We can perform basic statistical calculations to increase the number of information obtained from BP measurements by taking multiple readings. The arithmetic mean value can be used as an estimate for the true BP value. Since we have different results when making repeated BP measurements, we seek to know the width of the range of BP measurements. The spread of results informs us regarding the uncertainty of a BP measurement and standard deviation where the standard deviation is the basis of defining standard uncertainty denoted by *u*. The standard uncertainty is calculated as $u=\hat{\sigma }/\sqrt{n}$. In general, if an uncertainty estimate is acquired from the standard deviation of the repeated BP measurement results, it is referred to as Type A uncertainty estimate. All uncertainty estimates acquired without repeated BP measurement are called type B uncertainty estimates using assumed probability distributions, where the assumption can be made through either experience or information [11]. As mentioned above, since we do not know the true BP value, we need to know the reference value (BP obtained by the trained observers) for improving accuracy. This performance characteristics can be quantitatively expressed. Bias is the difference between the measured BP value obtained from multiple repeated BP measurements with the same sample and the reference value which is the considered the quantitative expression of the true value. These two values are combined into BP measurement uncertainty estimates and are considered to be a quantitative representation of accuracy.

We offer a combined measurement uncertainty for BPs through the bias, standard deviation etc. for the BP measurements. The combined standard uncertainty $u_{c}$ = $\sqrt{u_{\alpha }^{2}+u_{\beta }^{2}+u_{\gamma }^{2}}$, where $u_{\alpha }$ is the standard uncertainty as a random error, $u_{\beta }$ is the bias as a system error, and $u_{\gamma }$ is the maximum permissible error (against a mercury sphygmomanometer as a system error about ±1 mmHg [31]) which are the sources of uncertainty. We also provide a CI for expressing and evaluating uncertainty. The CI is a type of interval estimate, calculated from the statistics of the BP measurements, which might include the true BP value of an unknown population parameter. The CI is used as expanded uncertainty, i.e., $U=K\times u_{c}$, so that the CI of the measurand is acquired as $\bar{x}\pm U$ [4], where $\bar{x}$ denotes the mean of the measurand. If the measurand distribution close to converges toward a Gaussian distribution, the arithmetic mean value is given by $\bar{x}$, and the standard uncertainty is provided by standard deviation σ of this distribution. If K=2, then the CI is $\bar{x}\pm 2\sigma$, and the level of confidence climbs up to 95%.

### 4.2. CI Estimation Using the Bootstrap

The basic concept of this method is that it can utilize the uncertainty ranges of each BP measurement value to compute the maximum and minimum values for the CI. Thus, we provide the CI of the five BP estimates for each patient obtained from the EBRM algorithm, and explain the bootstrap principle of parameter estimation approach. The idea is to resample blood pressure hypotheses to produce many artificial blood pressure hypotheses, ${\hat{T}}^{*}=\left({\hat{t}}_{1}^{*},...,{\hat{t}}_{n}^{*}\right)$, based on *n* estimates obtained from an unknown distribution D(μ,σ) to compute a CI for $\hat{\mu }\left(T^{*}\right)$. Here, $\left[\hat{\mu },\hat{\sigma }\right]$ denotes the maximum likelihood estimate obtained using $\hat{T}=\left({\hat{t}}_{1},...,{\hat{t}}_{n}\right)$. Thus, when n→∞, we obtain a normal distribution given as $\hat{D}\left({\hat{\mu }}^{*},{\hat{\sigma }}^{*}|{\hat{T}}^{*}\right)≅N\left(\mu ,\sigma \right)$. In our work, we measure the CIs utilizing the bootstrap technique [14,22] which can be obtained using the BP estimates of the EBRM. We then obtain a matrix as follows:

(5) $$\begin{array}{c} M^{*}\left(i|{\hat{T}}_{i}^{S*}\right)=\left[\begin{array}{ccc} t_{1,1}^{*i} & \cdots & t_{1,B}^{*i}\\ \vdots & \ddots & \vdots \\ t_{n,1}^{*i} & \cdots & t_{n,B}^{*i} \end{array}\right], \end{array}$$

where Equation (5) is acquired as ${\hat{\mu }}_{i}^{*}+{\hat{\sigma }}_{i}^{*}\times \mathrm{RANDN}(n,B)$, we then vertically compute each column to obtain the average of each column as ${\hat{\mu }}_{b}^{s*}=1/n\sum_{j=1}^{n}t_{j,b}^{*i}$, where, s denotes SBP, and * indicates the resampled data obtained from the bootstrap technique. We, hence, do ascending sorts and the sorted BP estimate are given by ${\hat{\Xi }}^{s*}=\left({\hat{\mu }}_{1}^{s*},{\hat{\mu }}_{2}^{s*},\cdot \cdot \cdot ,{\hat{\mu }}_{B}^{s*}\right)$, assuming ${\hat{\mu }}_{\alpha }^{s*}$ is the 100α-th percentile of *B* bootstrap replications $\left({\hat{\mu }}_{1}^{s*},{\hat{\mu }}_{2}^{s*},\cdot \cdot \cdot ,{\hat{\mu }}_{B}^{s*}\right)$. We acquire the CI as ${\hat{\mu }}_{\mathrm{lower}}^{s*},{\hat{\mu }}_{\mathrm{upper}}^{s*}$ of the 1−2·α, from this bootstrap technique as $\left({\hat{\mu }}_{\alpha }^{s*},{\hat{\mu }}_{1-\alpha }^{s*}\right)$. Similar process is used to estimate the CI for DBP.

### 4.3. CI Estimation with the Monte Carlo Technique

In this work, we assume the expected parameters $E({\hat{\mu }}_{i}^{*},{\hat{\sigma }}_{i}^{*}|{\hat{T}}_{i}^{*})$ using a random variable based on the Monte Carlo technique [32]. Hence, we can create $\left\{{\hat{T}}_{1}^{*},...,{\hat{T}}_{n}^{*}\right\}$ to be independent and identically distributed (IID) random variables from the distribution of ${\hat{T}}^{*}$ using the estimated BP results using the EBRM and acquired their mean and standard deviation. Hence, we express a variance of ${\hat{\mu }}_{i}^{*}$ as $E\left[{\left({\hat{\mu }}_{i}^{*}-\mu \right)}^{2}\right]=\frac{{\sigma^{*}}^{2}}{n}$. The Monte-Carlo technique is a way to achieve an approximate error using sample values as $\frac{{\sigma^{*}}^{2}}{n}$. However, we do not know $\sigma^{2}$ exactly. Hence, we commonly use estimates of $\sigma^{2}$ given as

(6) $${\hat{\sigma }}^{*2}=\frac{1}{n-1}\sum_{i=1}^{n}{\left({\hat{T}}_{i}^{*}-{\hat{\mu }}_{i}^{*}\right)}^{2}.$$

A standard Gaussian distribution with a zero mean and unit variance has a probability density function as shown below:

(7) $$\rho \left(z\right)=\frac{1}{\sqrt{2\pi }}\exp^{-\frac{1}{2}z^{2}}.$$

Hence, a cumulative distribution function is calculated as

(8) $$\Phi \left(\alpha \right)=\int_{-\infty }^{\alpha }\rho \left(z\right)dz,\;\;\;-\infty <z<\infty .$$

Based on [32], if ${\hat{T}}_{i}^{*}$ is an IID with mean $\mu^{*}$ and variance $\sigma^{*2}>0$, then for all z∈R

(9) $$P\left(\sqrt{n}\frac{{\hat{\mu }}_{i}^{*}-\mu }{{\hat{\sigma }}^{*}}⩽z\right)⟶\Phi \left(z\right).$$

We then acquire the CI based on the theorem in [32] for μ but it requires that we know $\sigma^{*}$. $P(|{\hat{\sigma }}^{*}-\hat{\sigma }|>\epsilon )$ converges to zero for any ϵ>0. Hence, we can substitute *s* for $\hat{\sigma }$ where *s* is ${\hat{\sigma }}^{*}$. We then rewrite Equation (9) as

(10) $$\begin{array}{cc} P\left(|{\hat{\mu }}_{i}^{*}-\mu |⩾\frac{\lambda s}{\sqrt{n}}\right) & =P\left(\sqrt{n}\frac{{\hat{\mu }}_{i}^{*}-\mu }{s}⩽-\lambda \right)+P\left(\sqrt{n}\frac{{\hat{\mu }}_{i}^{*}-\mu }{s}⩾\lambda \right)\\ & \to \Phi (-\lambda )+(1-\Phi (-\lambda ))=2\Phi (-\lambda ). \end{array}$$

We can find a 5 % chance of non-coverage for a 95 % CI, and hence set 2Φ(−λ)=0.05, where λ>0. Therefore, we can denote as $\lambda =-\Phi^{-1}\left(.025\right)=\Phi^{-1}\left(.975\right)$ as

(11) $${\hat{\mu }}_{i}^{*}-\Phi^{-1}\left(.975\right)\frac{s}{\sqrt{n}}⩽\mu ⩽{\hat{\mu }}_{i}^{*}+\Phi^{-1}\left(.975\right)\frac{s}{\sqrt{n}},$$

where $\Phi^{-1}\left(.975\right)$ is 1.96. Thus, we acquire the CIs based on the estimated BP results using the EBRM.

## 5. Experimental Results

**Figure 1:**
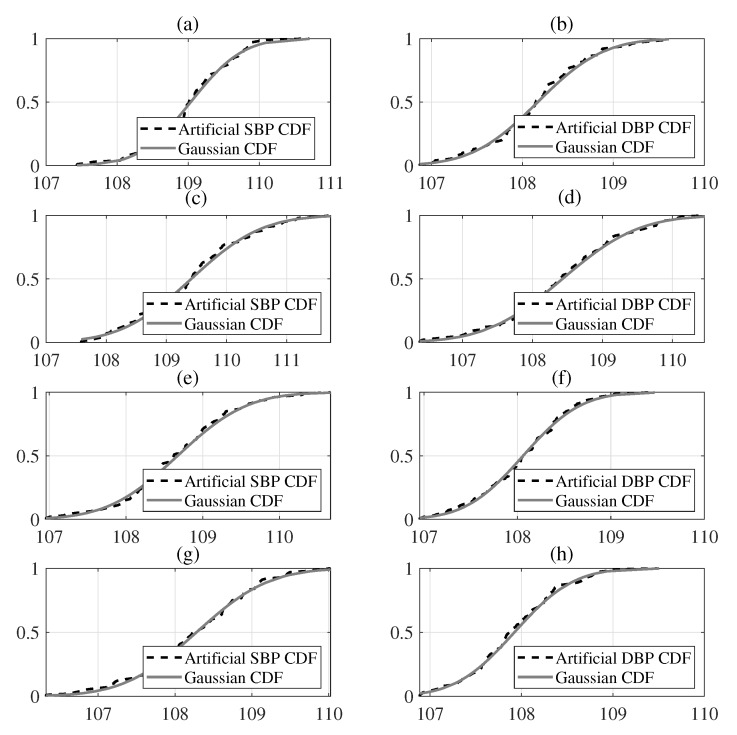
These figures denote the cumulative distribution function (CDF) of the selected artificial BP estimates from the parameter bootstrap approach with (*N* = 100) replicas based on the EBRM results, where *x*-axis denotes mmHg and *y*-axis denotes cumulative probability. Note that the plots (**a**) and (**b**) are the examples acquired from 5th subject, the plots (**c**) and (**d**) are the examples acquired from 6th subject, the plots (**e**) and (**f**) are the examples acquired from 7th subject, and the plots (**g**) and (**h**) are the examples acquired from 8th subject.

The BP measurements of volunteers were sequentially separated into training data and test data. Five sets of 300 BP measurements each of which taken from 60 volunteers were used as training data, and 125 measurements from 25 volunteers were used as validation data. This procedure was repeated so that each volunteer was included only once in the test process. In Table 3, an example result is shown in order to differentiate the artificial features from the original features. We summarized the parameters for the EBRM algorithm as in Table 4. As required by the ANSI/AAMI BP measurement protocol [16], the EBRM algorithm was evaluated to verify that the mean error (ME) is less than ±5 mmHg and that the standard deviation of error (SDE) is less than 8 mmHg, as shown in Table 5. Moreover, in accordance with the British Hypertension Society (BHS) protocol [1], the EBRM results were compared to the conventional methods, and the mean absolute error was evaluated for three groups of less than 5 mmHg, less than 10 mmHg and less than 15 mmHg, respectively (Table 5). If 60% of the mean absolute error of a BP measurement method is within 5 mmHg, 85% within 10 mmHg and 95% within 15 mmHg, the method is classified as class A. In Table 5, we provided the results of EBRM with respect to the uncertainty. Table 6 and Figure 1 show the results of statistical analysis through the Lilliefors function to evaluate the normality in terms of the artificial BP estimates for each subject.

## 6. Discussion

We confirmed that the uncertainty such as the bias for artificial data was extremely small, and that the standard error ${\hat{\sigma }}^{*}$, obtained from bootstrap technique, was smaller than $\hat{\sigma }$; hence, the bootstrap technique could be used as an efficient method to increase the number of samples of features. As a result, artificial features could be identified as being very close to actual features, and in addition, we found that the CI of artificial features subsumes the CI of all artificial and true features. We provided the standard uncertainty $u={\hat{\sigma }}^{*}/\sqrt{B(=100)}$, the combined uncertainty $u_{c}=\sqrt{\beta^{2}+u^{2}}$, and the expanded uncertainty $U=ku_{c}$ and all were easily calculated based on the approaches detailed in GUM [4] using the bias and standard error for artificial features (Table 3). The *u* and *U* in terms of the artificial features corresponded to very small values, which implies that there is little difference between artificial data and actual data.

Based on the BHS protocol [1] and as shown in Table 5, the mean absolute errors of the EBRM were 73.65% (≤5 mmHg), 93.88 % (≤10 mmHg), and 96.94 % (≤15 mmHg), respectively, for SBP and 83.06% (≤5 mmHg), 97.17 % (≤10 mmHg), and 99.76 % (≤15 mmHg), respectively, for DBP. Therefore, the EBRM obtained Class A designation for the measurement of SBP and DBP. We found that EBRM estimates are more accurate than BP estimates obtained using conventional methods. The accuracy of estimates obtained using EBRM was calculated by comparing them with estimates obtained using the stethoscope method in accordance with the ANSI/AAMI protocol [16]. The SDE of a device is generally considered more significant because unless there is a systematic bias, then MEs would be small even in the presence of large positive and negative errors. The proposed method met the ANSI/AAMI criteria and provided more accurate BP estimates compared with the conventional methods, as shown in Table 5. The SDE acquired using the EBRM was found to be 5.50 mmHg and 4.59 mmHg for the SBP and DBP, respectively. These results indicate superior performance compared to conventional algorithms. Therefore, we can conclude that the EBRM decreases the uncertainty related to measures such as the SDE of ME and increases the performance reliability. Moreover, the CIs for SBP and DBP obtained with ${\mathrm{EBRM}}_{\mathrm{BOOT}}$ were smaller than CIs obtained using the conventional methods, as shown in Table 6. We confirmed the difference between 1.7 mmHg and 1.0 mmHg of the EBRM and the conventional method [14] in the SDEs of CI for SBP and DBP although the CI results acquired from the EBRM are larger than those acquired from the ${\mathrm{DNN}}_{\varphi }$. The CIs in terms of the artificial BP values corresponded to very small values, which indicates that through the EBRM we successfully decreased the uncertainty with respect to a random error such as SDE and systematic error such as bias. We also provided the expanded uncertainty for the ${\mathrm{EBRM}}_{U}$ where ${\mathrm{EBRM}}_{U}$ was computed using the bias and standard error for artificial BP values according to the GUM recommendations [4] as shown in Table 6. We also obtained the CIs ${\mathrm{EBRM}}_{\mathrm{MC}}$ from the Monte Carlo technique based on the results of the EBRM as shown in Table 6. Specifically, the results of the CIs were smaller than those of the CIs obtained from the conventional methods.

The Lilliefors test is executed to verify the normality of each distribution, indicating that these distributions are very similar to Gaussian distributions [23]. We thus conducted the Lilliefors, correlation, kurtosis, and skewness tests based on the results of the EBRM in order to verify the normality for the individual BP measurements. As the number of *B* (=100) bootstrap replicas increased, the distribution of artificial BP measurements converged towards the Gaussian distribution. Hence, it was confirmed that the distribution of artificial BP measurements approaches the Gaussian distribution, as shown in Figure 1. The hypothesis for the average artificial BP measurement confirmed that *h* (=0.07) and *h* (=0.05) were almost zero for SBP and DBP estimates, which means that the null hypothesis was accepted at the 5% significance level. In addition, the null hypothesis could not be rejected because the *k* (=0.02) value was less than the threshold cv (=0.29) for SBP and DBP. Because our *p* (=0.33) and *p* (=0.36) test values were greater than 5% (=0.05) significant level for SBP and DBP, we affirmed the normality of the distribution of artificial BP measurements for each subject. In addition, kurtosis is also a measure of the population that determines how flat or peaked the probability distribution is compared to the Gaussian distribution, where the kurtosis of Gaussian distribution is 3. Thus, if the kurtosis value is greater than 3, distribution is heavy-tailed, and if the kurtosis value less than 3 the distribution is light-tailed. The kurtosis values for the artificial SBP and DBP measurements were 2.97 and 3.00, respectively, which indicates that the distributions of the artificial BP measurements were almost Gaussian. The skewness of the population is a measure of the horizontal symmetry for the distribution. Here, negative values show that the distribution is skewed to the left and positive values to the right whereas skewness of the Gaussian distribution is zero. We found that the symmetric distributions of the artificial BP data were 0.02 and −0.01, respectively, for the SBP and DBP, which means that they were nearly converging on the Gaussian distribution, as shown in Table 7.

Although we experimented with the BP data of 85 subjects based on the ANSI/AAMI protocol [31], this study was limited due to the small number of samples with relatively a small amount of participants. However, this limitation was resolved using artificial samples. Second, simultaneous measurements at the brachial and wrist locations were impossible because of the occlusion of the brachial artery by the upper arm cuff. Therefore, there was approximately 1.5 minutes’ separation between the BP measurements obtained using the automatic wrist BP monitor, and the reference measurements received simultaneously by the two trained nurses using the classic upper arm sphygmomanometer. This delay contributes to the measurement error because of the natural variation in SBP and DBP over even short periods. Third, although there are other measures of uncertainty for BP estimation, we focused on the random error (SDE) and systematic errors (i.e., ME, bias, and calibration errors) because we could only evaluate these errors in this study. We represented that CIs were short, but did not compensate for errors arising from the current setting of the experiment using oscillometric BP measurement.

## 7. Conclusions

In this paper, we propose a novel method using EBRM to measure uncertainty such as CIs, the standard deviation of error, bias, standard uncertainty, and expanded uncertainty for the SBP and DBP. We verify that the distribution of the artificial BP data is close to the Gaussian distribution, and identify similarities between the real data and the artificial data. The Lilliefors test is performed to investigate the normality of the artificial BP measurements for each subject. The main contribution of this work is that the accuracy and the stability of the blood pressure estimates are improved using the EBRM algorithm. We will carry out additional non-normality testing with a new subject population in the near future.

## Figures and Tables

**Table 1:** The process of blood pressure (BP) measurements for one subject.

Sequence (i)	Primary Nurse	Second Nurse	Average	OBPD
1	${\mathrm{SBP}}_{11}$ (=90) and ${\mathrm{DBP}}_{11}$ (=60)	${\mathrm{SBP}}_{21}$ (=90) and ${\mathrm{DBP}}_{21}$ (=60)	${\mathrm{SBP}}_{1}$ (=90) and ${\mathrm{DBP}}_{1}$ (=60)	${\mathrm{OBPD}}_{1}$
2	${\mathrm{SBP}}_{12}$ (=96) and ${\mathrm{DBP}}_{12}$ (=64)	${\mathrm{SBP}}_{22}$ (=94) and ${\mathrm{DBP}}_{22}$ (=64)	${\mathrm{SBP}}_{2}$ (=95) and ${\mathrm{DBP}}_{2}$ (=64)	${\mathrm{OBPD}}_{2}$
3	${\mathrm{SBP}}_{13}$ (=100) and ${\mathrm{DBP}}_{13}$ (=64)	${\mathrm{SBP}}_{23}$ (=98) and ${\mathrm{DBP}}_{23}$ (=64)	${\mathrm{SBP}}_{3}$ (=99) and ${\mathrm{DBP}}_{3}$ (=64)	${\mathrm{OBPD}}_{3}$
4	${\mathrm{SBP}}_{14}$ (=96) and ${\mathrm{DBP}}_{14}$ (=70)	${\mathrm{SBP}}_{24}$ (=96) and ${\mathrm{DBP}}_{24}$ (=70)	${\mathrm{SBP}}_{4}$ (=96) and ${\mathrm{DBP}}_{4}$ (=70)	${\mathrm{OBPD}}_{4}$
5	${\mathrm{SBP}}_{15}$ (=98) and ${\mathrm{DBP}}_{15}$ (=66)	${\mathrm{SBP}}_{25}$ (=96) and ${\mathrm{DBP}}_{25}$ (=66)	${\mathrm{SBP}}_{5}$ (=97) and ${\mathrm{DBP}}_{5}$ (=66)	${\mathrm{OBPD}}_{5}$

**Table 2:** The *p* are obtained from the artificial features utilizing the bootstrap technique *N* (=100), where *N* is a number of replication and *k*, *c*, and *h* are obtained from the Lilliefors-test function [24], where ${\mathrm{TSBP}}^{*}$ and ${\mathrm{TDBP}}^{*}$ denote target artificial SBP and DBP values, respectively.

Features/Parameters	*p*	*k*	*c*	*h*
TSBP*	0.500	0.060	0.089	0
TDBP*	0.470	0.090	0.089	0
MAP	0.500	0.050	0.089	0
AR	0.500	0.049	0.089	0
AE	0.420	0.063	0.089	0
EL	0.368	0.065	0.089	0
MA	0.352	0.065	0.089	0
${\mathrm{STD}}_{1}$	0.059	0.371	0.089	0
${\mathrm{STD}}_{2}$	0.063	0.500	0.089	0
MAPL	0.485	0.061	0.089	0

**Table 3:** An exemplary result (one subject) is represented to verify between the artificial features and original features for consistency and convergence [22].

Features/Parameters	$\hat{\mu }$	${\hat{\mu }}^{*}$	${\mathrm{CI}}_{L}^{*}$	${\mathrm{CI}}_{U}^{*}$	$\hat{\sigma }$	${\hat{\sigma }}^{*}$	$\beta ({\hat{\mu }}^{*}\left(\cdot \right))$	*u*	*U*
TSBP	93.20	93.35	91.56	95.84	2.39	1.03	0.152	0.103	±0.367
TDBP	59.80	59.90	57.69	61.93	2.17	0.95	0.101	0.095	±0.278
MAP	0.311	0.312	0.253	0.365	0.057	0.027	0.001	0.0027	±0.0057
AR	0.494	0.496	0.451	0.533	0.045	0.020	0.002	0.002	±0.0052
AE	0.065	0.066	0.057	0.077	0.012	0.005	0.001	0.0005	±0.0022
EL	0.236	0.236	0.231	0.242	0.006	0.002	0.000	0.0002	±0.0005
MA	0.166	0.165	0.141	0.194	0.026	0.011	−0.001	0.001	±0.002
${\mathrm{STD}}_{1}$	0.150	0.151	0.101	0.204	0.054	0.025	0.001	0.003	±0.03
${\mathrm{STD}}_{2}$	0.184	0.183	0.133	0.228	0.047	0.022	−0.001	0.002	±0.005
MAPL	0.391	0.390	0.360	0.416	0.031	0.013	−0.001	0.001	±0.003

**Table 4:** Summarized parameters [17,28] of the ensemble-based recursive methodology (EBRM) algorithm, where 12 denotes the dimension of input vector, 2 is the number of output units (namely, the target vector [SBP and DBP] dimensions), and 32 is the number of hidden unit.

Number of the Units:	[(12,(32),(32), (32), 2)]
Dimension of feature	12
Dimension of target	2
Number of hidden layers	3
Number of hidden unit on the layers	32
Number of sample over original feature	5
Number of sample over each artificial feature	100
Number of epoch in the pre-training	10 to 50
Number of epoch in the fine-tuning	10 to 50
Learning rate for weight	0.001
Learning rate for biases of visible units	0.01
Learning rate for biases of hidden units	0.01
Momentum rate	0.9
Activation type	logistic function
Initial weights and biases	randomly between (−1, 1)

**Table 5:** Evaluating of the EBRM algorithm through the British Hypertension Society (BHS) and Association for the Advancement of Medical Instrumentation (AAMI) protocols utilizing the results of maximum amplitude algorithm (MAA), neural network (NN), support vector regression (SVR) [33], deep neural network (DNN), ${\mathrm{DNN}}_{\varphi }$ and EBRM on (5×85=425) measurements, where ${\mathrm{DNN}}_{\varphi }$ denotes the Adaboost with DNN model.

Methods	SBP			DBP			SBP/DBP	SBP	DBP
	Mean Absolute Difference (%)			Mean Absolute Difference (%)			BHS	AAMI	
	≤5 mmHg	≤10 mmHg	≤15 mmHg	≤5 mmHg	≤10 mmHg	≤15 mmHg	Grade	ME(SDE)	ME(SDE)
MAA	47.06	85.88	96.47	56.47	88.24	97.65	C/B	0.07 (9.28)	−0.89 (7.76)
NN	53.88	85.65	95.53	66.12	94.12	98.82	B/A	0.25 (7.48)	−0.22 (6.80)
SVR	62.59	86.12	95.53	74.12	93.65	96.94	A/A	0.10 (7.15)	−0.34 (6.45)
DNN	69.18	88.71	95.53	76.24	93.17	98.12	A/A	0.02 (6.44)	0.11 (5.24)
${\mathrm{DNN}}_{\varphi }$	71.06	90.82	95.53	81.18	96.24	99.29	A/A	−0.05 (5.72)	0.05 (4.70)
EBRM	73.65	93.88	96.94	83.06	97.17	99.76	A/A	0.02 (5.50)	0.18 (4.59)

**Table 6:** Comparison of CIs between the proposed EBRM and conventional methods, where n (=85) denotes the number of subject, L and U denote the lower and upper limits, respectively.

BP (mmHg)	SBP (SDE)	DBP (SDE)	SBP L (SDE)	SBP U (SDE)	DBP L (SDE)	DBP U (SDE)
n (=85)	95%CI	95%CI				
${\mathrm{MAA}}_{\mathrm{ST}}$ [14]	13.2 (8.0)	9.4 (5.8)	106.7 (14.3)	120.2 (16.5)	62.4 (10.4)	71.7 (11.0)
${\mathrm{MAA}}_{\mathrm{GUM}}$ [14]	13.9 (7.9)	10.0 (5.4)	106.4 (14.3)	120.5 (16.4)	62.0 (10.4)	72.1 (10.9)
${\mathrm{PMAE}}_{\mathrm{NPB}}$[14]	2.8 (3.3)	1.7 (2.4)	112.4 (13.9)	115.7 (14.1)	66.7 (10.5)	68.2 (9.9)
${\mathrm{DNN}}_{\mathrm{BOOT}}$	5.5 (1.3)	4.2 (0.8)	107.4 (12.7)	113.0 (12.6)	64.5 (8.3)	68.6 (8.4)
${\mathrm{DNN}}_{\varphi (\mathrm{BOOT})}$	4.8 (1.5)	4.2 (0.9)	107.3 (12.7)	112.1 (12.8)	65.1 (8.2)	69.3 (8.8)
${\mathrm{EBRM}}_{\mathrm{BOOT}}$	3.1 (2.9)	3.2 (2.7)	107.9 (13.9)	111.0 (13.4)	65.5 (9.4)	68.7 (9.0)
${\mathrm{EBRM}}_{\mathrm{MC}}$	1.4 (0.4)	1.2 (0.4)	107.8 (12.8)	109.2 (13.4)	65.0 (9.2)	66.3 (9.4)
${\mathrm{EBRM}}_{U}$	6.6 (2.7)	6.8 (3.3)	105.7 (12.8)	112.3 (13.4)	63.8 (9.3)	70.6 (9.3)

**Table 7:** Summary of the Lilliefors and Normality tests for SBP and DBP (85 subjects).

Tests	Lilliefors Test			Normality Test		
α **(=0.05)**	**h (std)**	**p (std)**	**k (std)**	**cv (std)**	**kurtosis (std)**	**skewness (std)**
SBP	0.07 (0.27)	0.33 (0.18)	0.02 (0.005)	0.29 (0.00)	2.97 (0.17)	0.02 (0.08)
DBP	0.05 (0.21)	0.36 (0.16)	0.02 (0.005)	0.29 (0.00)	3.00 (0.18)	−0.01 (0.08)
